# Gaseous environment modulates volatile emission and viability loss during seed artificial ageing

**DOI:** 10.1007/s00425-021-03620-5

**Published:** 2021-04-17

**Authors:** Biao Han, Vincent Fernandez, Hugh W. Pritchard, Louise Colville

**Affiliations:** 1 Shandong Forest Germplasm Resources Center, Ji’nan City, China; 2 Imaging and Analysis Centre, Natural History Museum, Cromwell Road, London, UK; 3 Department of Comparative Plant and Fungal Biology, Royal Botanic Gardens, Kew, Wakehurst Place, Ardingly, UK

**Keywords:** Anoxia, Longevity, Oxygen, Seed storage, Silica gel

## Abstract

**Main conclusion:**

Modulation of the gaseous environment using oxygen absorbers and/or silica gel shows potential for enhancing seed longevity through trapping toxic volatiles emitted by seeds during artificial ageing.

**Abstract:**

Volatile profiling using non-invasive gas chromatography–mass spectrometry provides insight into the specific processes occurring during seed ageing. Production of alcohols, aldehydes and ketones, derived from processes such as alcoholic fermentation, lipid peroxidation and Maillard reactions, are known to be dependent on storage temperature and relative humidity, but little is known about the potential modulating role of the gaseous environment, which also affects seed lifespan, on volatile production. Seeds of *Lolium perenne* (Poaceae), *Agrostemma githago* (Caryophyllaceae) and *Pisum sativum* (Fabaceae) were aged under normal atmospheric oxygen conditions and in sealed vials containing either oxygen absorbers, oxygen absorbers and silica gel (equilibrated at 60% RH), or silica gel alone. Seeds of *A. githago* that were aged in the absence of oxygen maintained higher viability and produced fewer volatiles than seeds aged in air. In addition, seeds of *A. githago* and *L. perenne* aged in the presence of silica gel were longer lived than those aged without silica, with no effect on seed moisture content or oxygen concentration in the storage containers, but with silica gel acting as a volatile trap. These results indicate that the use of inexpensive oxygen absorbers and silica gel could improve seed longevity in storage for some species and suggests a potential, and previously unidentified, role for silica gel in ultra-dry storage.

**Supplementary Information:**

The online version contains supplementary material available at 10.1007/s00425-021-03620-5.

## Introduction

The effects of temperature and moisture on seed longevity in storage have been well characterised, and in general, the life span of orthodox, desiccation tolerant, seeds increases as temperature and moisture content decrease, within certain limits (Dickie et al. 1990; Ellis et al. 1990). Although, this does not hold true for all species, particularly under ultra-dry conditions (Ballesteros and Walters 2011). The international standards for long-term ex situ seed storage (FAO 2014) recommend that seeds are stored at low temperature (ca. -18 °C) after drying to low relative humidity (ca. 15% RH). Besides temperature and moisture content, gaseous environment may affect seed life span (Roberts and Abdalla 1968; Ibrahim and Roberts 1983; Ibrahim et al. 1983; Tompsett 1983). Research into the process of seed deterioration during storage has indicated the involvement of reactive oxygen species and accumulation of oxidative damage to cellular macromolecules e.g. lipids, proteins and nucleic acids, which eventually leads to loss of cell function and seed death (Bailly 2004; Rajjou et al. 2008; Kranner et al. 2011). For this reason, much of the focus of research into the effect of the gaseous environment on seed storage has been on oxygen. Comparisons of hermetic storage and open storage at a range of seed moisture contents showed that longevity was higher in hermetic storage for seeds with low moisture content, and the difference is likely due to the limited availability of oxygen in hermetic storage (Ellis and Hong 2007).

The life span of seeds in storage varies greatly between species (Walters et al. 2005; Probert et al. 2009) and also between seed lots of the same species (Nagel et al. 2009), but the characteristics underpinning seed lifespan have yet to be determined. A recent analysis of seed life span under a range of conditions has shown that most species may produce seeds that are comparatively short-lived i.e. more than half of species have seed life spans < 20% of the longest lived under the same conditions (Colville and Pritchard 2019). Predicting how long seeds will survive in ex situ storage is difficult. Viability equations for the estimation of storage life at a range of moisture and temperature conditions have been defined but require determination of species-specific temperature and moisture constants (Ellis and Roberts 1980). Therefore, monitoring of seed viability during storage is a critical component of the management of ex situ seed collections and requires optimisation of testing intervals to minimise wastage of seeds by testing too frequently or conversely reduce the risk of not detecting deterioration of seed collections by testing too infrequently. The standard approach for monitoring seed viability is germination testing, which is time-consuming and destructive (Hay and Whitehouse 2017). Techniques for the rapid, and ideally non-destructive screening of seed viability have been the focus of research (e.g. Costanzo et al. 2008; Kranner et al. 2010; Xin et al. 2013). However, there is not yet a single approach that can be universally applied for monitoring seed viability of diverse species. One technique that has received attention is the analysis of volatile metabolites evolved by seeds during storage. These metabolites accumulate in sealed storage containers and can be detected using gas chromatography–mass spectrometry (GC–MS). A wide range of volatile compounds have been detected during seed storage, among them ethanol, methanol, acetaldehyde and acetone are frequently reported to accumulate during storage of seeds of a number of species including *Pisum sativum* (Zhang et al. 1995; Colville et al. 2012); *Lathyrus pratensis* and *Cytisus scoparius* (Colville et al. 2012); *Glycine max* (Zhang et al. 1994, 1995; Lee et al. 2001); *Phaseolus vulgaris* (Lee et al. 2001); *Lactuca sativa* (Zhang et al. 1994, 1995; Mira et al. 2010, 2016); *Carum carvi* and *Eruca vescaria* (Mira et al. 2016); *Oryza sativa* (Zhang et al. 1994, 1995); *Xanthium pennsylvanicum* (Zhang et al. 1995); *Brassica napus* (Buckley and Buckley 2009); *Helianthus annuus* and *Daucus carota* (Zhang et al. 1994).

In addition to its potential as a non-invasive technique for monitoring seed viability, seed volatile profiling also provides an insight into the processes taking place within seeds during storage (Mira et al. 2016). The volatile compounds released by seeds of three legume species (*C. scoparius*, *L. pratensis* and *P. sativum*) during storage are reported to derive from a number of processes including respiration, lipid peroxidation and Maillard reactions (Colville et al. 2012). The occurrence of these reactions is highly dependent on the moisture content of the seeds and temperature (Wettlaufer and Leopold 1991), which will determine viscosity of the cytoplasm and molecular mobility therein, i.e., whether the seed cytoplasm is in a glassy state (Sun 1997). The high viscosity of intracellular glasses minimises molecular mobility and therefore limits the occurrence of chemical reactions that contribute to seed deterioration during storage (Buitink et al. 2000). Mitochondrial respiration does not occur in seeds stored at < 75% RH, but glycolysis has been shown to occur at a slow rate leading to production of ethanol and acetaldehyde, which have been detected from *G. max* and *P. vulgaris* seeds stored between 12 and 75% RH, via alcoholic fermentation (Lee et al. 2001). Mira et al. (2010, 2016) reported that at > 30% RH volatile organic compounds (VOCs) emitted by seeds of *C. carvi*, *E. vescaria* and *L. sativa* derived from fermentation-type reactions, and at < 30% RH, there was a switch towards peroxidation-type reactions. However, viability was more strongly correlated with emission of fermentation products and there was no correlation between ageing and emission of lipid peroxidation products.

The presence of oxygen may also influence the types of reactions that take place during seed storage, particularly those related to respiration and oxidative processes, e.g., lipid peroxidation. In metabolically active plant tissues, a reduction in oxygen concentration leads to a rapid inhibition of respiration and downregulation of the Krebs cycle and glycolysis to reduce oxygen consumption and avoid internal anoxia (Geigenberger 2003). Developing seeds of *Vicia faba* and *P. sativum* have been shown to have very low levels of oxygen of around 2–3%, which limits the rate of metabolism, but anoxia and induction of fermentation were not detected (Rolletschek et al. 2002). The internal oxygen concentration of mature, dry pea seeds was reported to be in equilibrium with the atmosphere, but rapidly falls during imbibition due to the onset of respiration and low oxygen diffusion through the seed coat, leading to fermentation (Rolletschek et al. 2009). Despite the potential benefits to seed life span of storage under hypoxia or anoxia (Groot et al. 2015), there have been few studies of the biochemical effects of reduced oxygen levels during seed storage (Rutzke et al. 2008).

In this study, the influence of oxygen in the gaseous storage environment on the life span of seeds of three species, *Agrostemma githago*, *Lolium perenne* and *P. sativum*, during artificial ageing at 60% RH and 50 °C was investigated. The species were chosen to represent a diverse range of phylogeny, seed size and structure, particularly embryo: seed ratio, to determine the variation in response to ageing in modified gaseous environments. Oxygen absorbing sachets were used to generate an anoxic storage environment (< 0.1% oxygen), and comparison was made with seeds aged in ambient atmospheric conditions, and with seeds aged in the presence of sachets of silica gel both alone and in combination with O_2_ absorbers. The silica gel had been equilibrated at 60% RH and was included to control for a potential moisturising effect of the oxygen absorbers. Seed deterioration during artificial ageing was monitored by germination testing and assessment of seedling growth. In addition, volatile profiling of the headspace of the storage containers using GC–MS was conducted to gain an insight into the processes taking place within seeds during ageing in the presence and absence of oxygen. To assess the potential toxicity of the most abundant volatile metabolites which accumulate in the headspace of storage containers during ageing, seeds of *P. sativum* were subjected to 10 d of artificial ageing in the presence of exogenously applied pure volatile compounds. The adsorption of these volatile compounds by O_2_ absorbers, silica gel and the seeds themselves was determined by solid phase microextraction (SPME)-GC–MS analysis.

## Materials and methods

### Seed material

Seeds of *L. perenne* L. and *A. githago* L. were obtained from Emorsgate Seeds (King's Lynn, Norfolk, UK) and *P. sativum* ‘Meteor’ seeds were purchased from CN Seeds Ltd (Pymoor, UK). All seeds were stored at 15% RH and 15 °C prior to use.

### Seed morphology

**Fig. 1:**
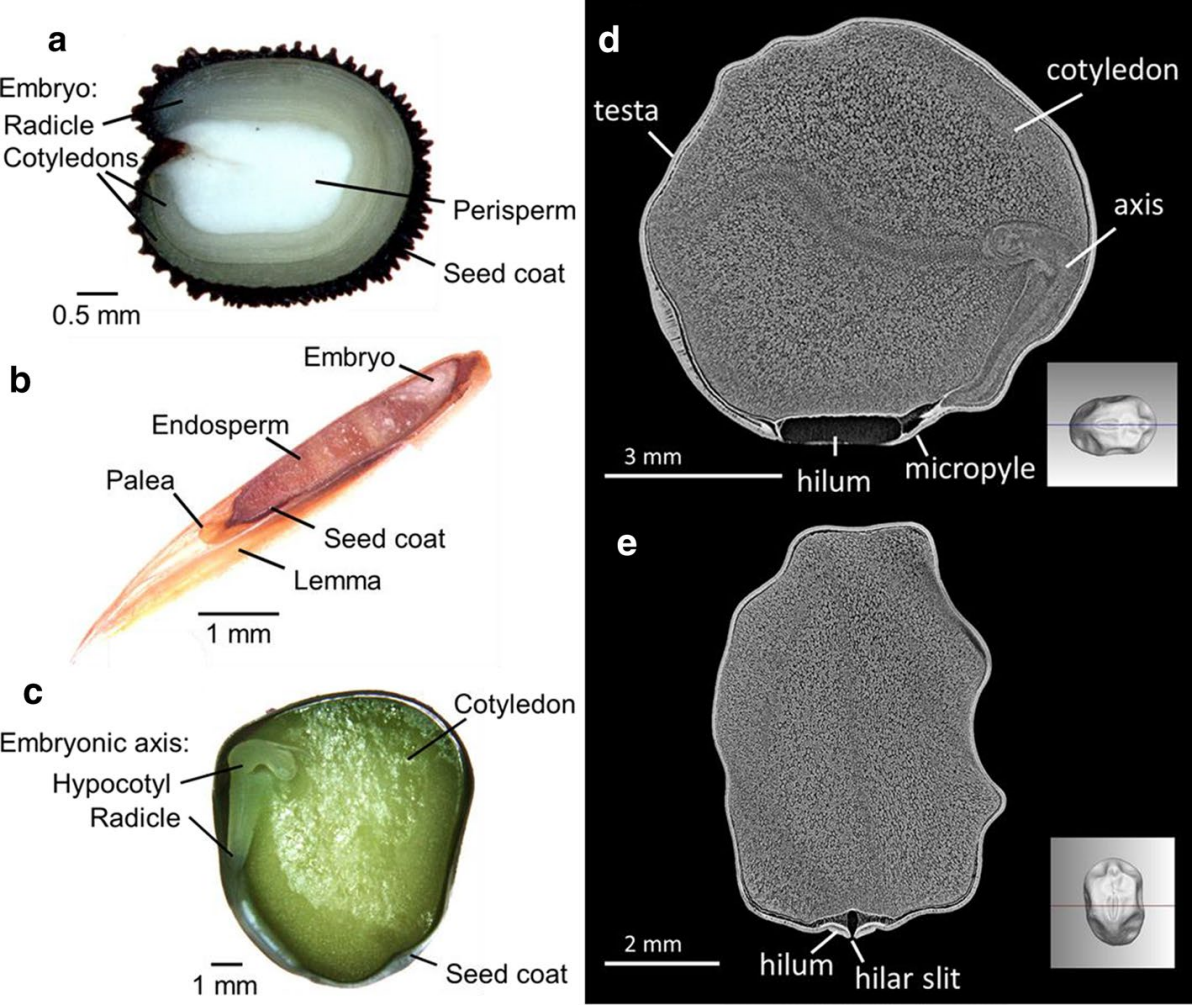
Longitudinal sections of imbibed seeds of *Agrostemma githago* (**a**), *Lolium perenne* (**b**) and *Pisum sativum* (**c**) showing the differences in size and structure between the three species. X-ray micro-computed tomography images of simulated thin sections of a dry *Pisum sativum* seed showing longitudinal (**d**) and transverse (**e**) sections through the hilum

Seeds were imbibed for 24 h, frozen in Optimal Cutting Temperature compound and cut using a CM3050 cryostat (Leica Microsystems, Wetzlar, Germany) at − 20 °C. The seeds were then imaged using a digital microscopy camera (Zeiss Axiocam) mounted on a stereomicroscope (Zeiss Stemi SV11). The images were taken using Axiovision version 3.1 (Carl Zeiss, Jena, Germany), see Fig. 1.

### X-ray micro-computed tomography

A dry (15% RH) seed of *P. sativum* was characterised with a Zeiss Xradia 520 Versa CT system (Carl Zeiss Microscopy) using X-ray micro-computed tomography (XCT). The instrument was set up to obtain an appropriate transmission of X-rays through the sample. The experimental conditions consisted of voltage of 60 kV and current of 83.3 µA (power of 5 W); no filter. Data were recorded with an indirect detector (scintillator screen, 0.39 × optical lens, Andor iKon-L Charge-coupled device camera with 2048 × 2048 active pixels of 13.5 × 13.5 µm). The acquisition consisted of 2401 projections of 6 s each. The combined magnification from the X-ray conical beam geometry (Source-Object distance: 15.02 mm; Object-Detector distance: 110.00 mm) and the specifications of the indirect detector generated data with an isotropic voxel size of 4.14 µm. The tomographic acquisition and reconstruction were performed using Zeiss Scout-and-Scan software (Carl Zeiss Microscopy). The reconstruction first consisted of a 32-bit volume and was later converted to a 16-bit stack of tiff files using Zeiss XRController software, see Fig. 1.

### Seed oil content

Seed oil content was quantified by time domain-nuclear magnetic resonance (TD-NMR) using a Bruker mq20 minispec, with a 0.47 T magnet (20 MHz proton resonance frequency) operating at 40 °C and a 10 mm probe assembly. Samples were analysed using a calibration with seeds of *H. annuus* of known oil content, where the method consisted of the acquisition of 16 scans with a recycle delay of 2 s. The method was modified from Borisjuk et al. (2011). Seed samples for each species were divided into five replicates of 100 mg and placed in 10 mm diameter NMR vials. For *P. sativum*, seeds were first cut into small, irregular fragments with a scalpel. 100 mg of the resulting seed fragments were then transferred to the NMR vials at random.

### Seed ageing treatments

**Fig. 2:**
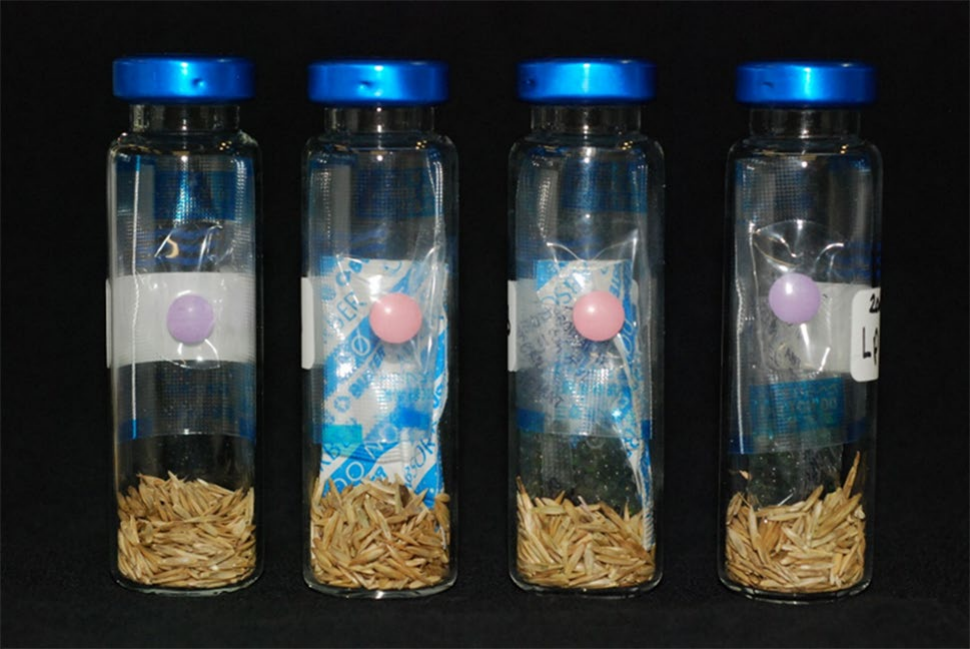
Photograph showing the experimental set up. 20 mL glass headspace vials were filled with 1 g of seeds (seeds pictured are *Lolium perenne*) which had been equilibrated to 60% RH. Control vials (left) contained just seeds and an oxygen indicating tablet, which was blue/purple in the presence of oxygen (> 0.5%) and turned to pink when oxygen levels were < 0.1%. Other vials (second from left to right) contained seeds, an oxygen indicating tablet and either a 20 cc oxygen absorber (O_2_ absorber), a 20 cc oxygen absorber and a silica gel sachet (O_2_ absorber + silica), or a silica gel sachet which had been pre-equilibrated to 60% RH (silica). All vials were sealed with PTFE-lined crimp seals

Seeds were aged under the conditions used for *P. sativum* in previous experiments (Kranner et al. 2006; Colville et al. 2012; Chen et al. 2013) Seeds were equilibrated over non-saturated lithium chloride solutions at 60% RH and 20 °C. The RH of the lithium chloride solution and the equilibrium RH of the seeds was determined using a hygrometer (HygroPalm, Rotronic Instruments Ltd, Crawley, UK). Following equilibration, 1 g of seeds were placed into 20 mL glass vials containing an oxygen indicating tablet (RP System Indicating eye, Conservation by Design, Milton Keynes, UK), which turn pink when oxygen concentrations are below 1%, and either a 20 cc oxygen absorbing sachet (O-Buster FT-type, Hsiao Sung Non-Oxygen Chemicals, Taichung City, Taiwan), a sachet of silica gel which had been pre-equilibrated at 60% RH, or an oxygen absorbing sachet and silica gel. The O-Buster FT-type oxygen absorbers are a self-reacting type, containing moistened zeolites to provide the water required for the oxidation of iron powder. The oxygen absorbers are effective at low RH and do not require additional water (from the sample) to be activated. Control vials contained only seeds and an oxygen indicating tablet (Fig. 2). The vials were hermetically sealed and placed in an oven at 50 °C. Vials were removed from the oven at intervals during artificial ageing (after 0, 5, 10, 15 and 20 days for *P. sativum* and *L. perenne*; after 0, 1, 2, 3 and 4 days for *A. githago*). The length of ageing was determined in trial experiments (unpublished). Three replicate vials were used for each treatment and ageing time point.

### Volatile profiling

Volatiles accumulated in the headspace of the vials were sampled using solid phase microextraction (SPME) and analysed using gas chromatography–mass spectrometry (GC–MS; Colville et al. 2012; Paulsen et al. 2013). Prior to headspace sampling, vials were incubated at room temperature for 24 h to allow the headspace to equilibrate following the transfer from 50 °C. Volatile analysis was performed using automated SPME with a 75 μm Carboxen polydimethylsiloxane (PDMS) fibre (Supelco, Bellefonte, PA, USA) and an extraction time of 30 min at 30 °C followed by 5 min of desorption in the GC injector port at 240 °C. The volatiles were separated using GC (Thermo Finnigan Trace GC Ultra; Thermo Fisher Scientific, Waltham, MA, USA) on a FAMEWAX column (30 m length, 0.25 mm internal diameter, 0.25 μm film thickness; Restek, Bellefonte, PA, USA) running a temperature program (3 min hold at 35 °C, 3 °C min^−1^ to 60 °C, 10 °C min^−1^ to 220 °C and 1 min hold; helium carrier gas at constant flow rate of 1 mL min^−1^). The volatiles were detected using mass spectrometry (MS; Thermo Finnigan Trace DSQ; ionisation energy 70 eV and scan frequency range *m*/*z* 10–350 per 0.5 s). The volatile compounds were identified by spectral library matching with the NIST (National Institute of Standards and Technology, Gaithersburg, MD, USA) mass spectral database. The identities of the volatile compounds were confirmed and quantified through comparison with analytical standards (Sigma Aldrich). Standards were prepared as a mixture containing 24 compounds and diluted with dimethyl sulfoxide to produce nine different concentrations, which ranged from 2.5 to 100 μM for ethyl acetate (lowest concentration standard) to 0.9–35 mM for acetic acid (highest concentration standard). Aliquots of 10 μL were placed into 20 mL headspace vials, which were sealed and analysed using SPME–GC–MS as described above.

### Moisture content, germination and seedling growth evaluation

Subsequently, seed moisture content was determined gravimetrically following oven drying at 103 °C for 17 h to assess the effect of the treatments, and particularly the presence of oxygen absorbers and/or silica gel on seed moisture content. In addition, the equilibrium relative humidity (eRH) of silica gel sachets was measured using a hygrometer probe (Rotronic). Silica gel sachets were removed from the sample vials containing seeds following ageing, and two sachets were immediately sealed in a 10 mL vial and RH was recorded after 30 min at 20 °C. Seed viability was assessed by germination testing. For each replicate (*n* = 3), 10 (*P. sativum*) or 20 (*L. perenne* and *A. githago*) seeds were sown in 10 cm diameter Petri dishes on 1% (w/v) agar. Only 10 seeds were used per replicate for *P. sativum* due to the size of the seeds (1 g of seeds was used per replicate for the ageing experiments, which is ca. 13–14 seeds for *P. sativum*). *L. perenne* and *P. sativum* seeds were incubated at a constant temperature of 20 °C with a 12 h photoperiod, and *A. githago* seeds were incubated at an alternating temperature of 20/10 °C with a 12 h photoperiod, as per the germination conditions for these species recorded in Royal Botanic Gardens, Kew’s Seed Bank Database. Germination was scored daily as the emergence of a radicle > 2 mm in length (hereafter referred to as total germination). After 14 d (*P. sativum*), 21 d (*L. perenne*) and 28 d (*A. githago*), normal seedling development was assessed to determine that seedlings showed vigorous and balanced growth of roots and shoots, and had potential to develop into healthy plants. Seedlings were photographed and root and shoot length was measured using Image J (Schneider et al. 2012).

### Volatile treatments

To assess the effects of individual volatile compounds on seed viability during ageing, *P. sativum* seeds were aged for 10 d at 60% RH and 50 °C in the presence of either 1.25 μmol ethanol, 3 μmol methanol, 0.6 μmol acetic acid, 0.85 μmol 2-propanol, 90 nmol acetone or 40 nmol acetaldehyde. The concentrations of the compounds were in the range of those measured in the headspace of control *P. sativum* seeds following artificial ageing for 15 d. The pure compounds were pipetted individually into 2 mL glass vials. Each 2 mL vial was immediately placed inside a 20 mL glass vial containing 1 g of *P. sativum* seeds. Three replicates were used for each treatment. Following ageing, the volatile accumulation in the headspace was assessed by SPME–GC–MS and germination and root and shoot length determined as described above. For comparison with the previous experiment, control vials containing only seeds, vials containing seeds and an oxygen absorbing sachet, and vials containing seeds and silica gel were set up and volatile accumulation, germination and seedling growth were determined in unaged (0 d) and aged (10 d) seeds.

To determine the adsorption of volatile compounds by silica gel, oxygen absorbing sachets, *A. githago* seeds, and *L. perenne* seeds the same amounts of pure compounds as used above were placed into 2 mL glass vials and added to 20 mL glass vials either alone (controls) or along with sachets of silica gel (equilibrated to 60% RH), oxygen absorbing sachets or 1 g of *A. githago* or *L. perenne* seeds. The vials were sealed and placed at 50 °C for 10 d following which volatile analysis was performed using SPME–GC–MS. Three replicates were performed for each treatment.

### Statistical analysis

One-way ANOVA followed by Dunnett’s post hoc test was performed using Genstat (Version 12, VSN International, 2011) to test for significant (*P* ≤ 0.05) differences between treatments and the control for each species. Principal component analysis (PCA) was performed on 26 variables (total germination, normal germination, emission rate of individual VOCs and total VOC emission rate) for all species combined, using XLSTAT. Data were automatically standardised, and Pearson correlation coefficients were computed by the software (Suppl. Table S1).

## Results

### Seed morphology

The seeds of the three species differed in size and morphology. *L. perenne* are the smallest, with an average 1000 seed weight of 2.0 g, compared to 12.0 g for *A. githago* and 139.9 g for *P. sativum* (Royal Botanic Gardens Kew 2020). *A. githago* has a peripheral embryo, which occupies around half of the seed volume, and surrounds the perisperm tissue. The *L. perenne* caryopsis consists of a small lateral embryo and abundant endosperm enclosed in a thin seed coat with lemma and palea attached. *P. sativum* has a bent embryo, which occupies the total seed volume with the cotyledons forming the storage reserves for the seed instead of endosperm tissue (Fig. 1; Martin 1946). Micro-computed tomography of a dry *P. sativum* seed showed more detail of the dry seed structure, particularly the hilum, which was open. Intercellular spaces could be seen in the cotyledons, but were not visible in the axis, in which cell density appeared to be higher (Fig. 1d).

### Effect of storage environment on germination and seedling growth during artificial ageing

The moisture content of the unaged control seeds was 12.7 ± 0.38%, 10.8 ± 0.33% and 11.9 ± 0.07% for *A. githago*, *L. perenne* and *P. sativum*, respectively. Moisture content did not change during ageing and was also unaffected by the presence of O_2_ absorbers or silica gel (Table 1). Likewise, the eRH of silica gel sachets was unaffected by the ageing treatments, and there was no difference in the eRH of silica gel which had been incubated with seeds or with seeds and O_2_ absorbers (Table 1). The oxygen indicators sealed within each vial remained pink throughout the duration of the ageing time courses in all vials containing O_2_ absorbers, showing that the oxygen levels were below 0.1%. In vials without O_2_ absorbers, the oxygen indicators were blue, with no change in colour to indicate any changes in oxygen levels (Fig. 2).

**Table 1:** Moisture content of seeds of *Agrostemma githago*, *Lolium perenne* and *Pisum sativum*, and equilibrium relative humidity (eRH) of silica gel sachets following equilibration at 60% RH (at 20 °C) and incubation at 50 °C

Aged (d)	Seed moisture content (% _FWB_)				Silica eRH (%)	
	Control	O_2_ ab	Si	O_2_ ab. + Si	Si	O_2_ ab. + Si
*A. githago*						
0	12.69 (0.384)	10.98 (0.944)	12.11 (0.123)	12.36 (0.258)	59.4	58.3
1	12.21 (0.081)	11.83 (0.036)	12.08 (0.184)	11.63 (0.153)	58.1	57.4
2	11.77 (0.088)	11.48 (0.203)	11.31 (0.128)	11.28 (0.096)	57.6	57.3
3	11.75 (0.582)	11.51 (0.228)	11.47 (0.199)	11.73 (0.403)	58.5	59
4	11.19 (0.089)	11.42 (0.324)	11.82 (0.190)	12.31 (0.670)	59.3	58.6
*L. perenne*						
0	10.84 (0.331)	9.90 (1.053)	11.43 (0.178)	10.91 (0.708)	58.7	58.4
5	10.74 (0.350)	10.70 (0.451)	10.55 (0.087)	10.83 (0.561)	59.1	59
10	10.88 (0.136)	10.41 (0.179)	9.91 (0.110)	10.19 (0.056)		
15	10.59 (0.223)	10.14 (0.311)	10.40 (0.291)	10.94 (0.672)	59	57.8
20	11.04 (0.041)	10.59 (0.026)	10.64 (0.025)	10.33 (0.057)	60.2	59.3
*P. sativum*						
0	11.92 (0.074)	12.13 (0.160)	12.02 (0.052)	11.87 (0.120)	58.7	58.6
5	11.00 (0.024)	11.02 (0.069)	11.06 (0.079)	11.05 (0.011)	59.8	59.5
10	11.62 (0.037)	11.60 (0.091)	11.67 (0.106)	11.60 (0.058)		
15	11.45 (0.030)	11.36 (0.021)	11.40 (0.059)	11.43 (0.130)	60.8	58.9
20	11.35 (0.043)	11.21 (0.116)	11.32 (0.041)	11.18 (0.179)	61.5	59.6

Moisture content is expressed on a fresh weight basis. Values represent mean (*n* = 3) with SE shown in parentheses. There were no significant differences in seed moisture content between storage treatments. Silica eRH was measured using a hygrometer. A single measurement was performed per treatment using silica gel sachets from two of the three replicates

**Fig. 3:**
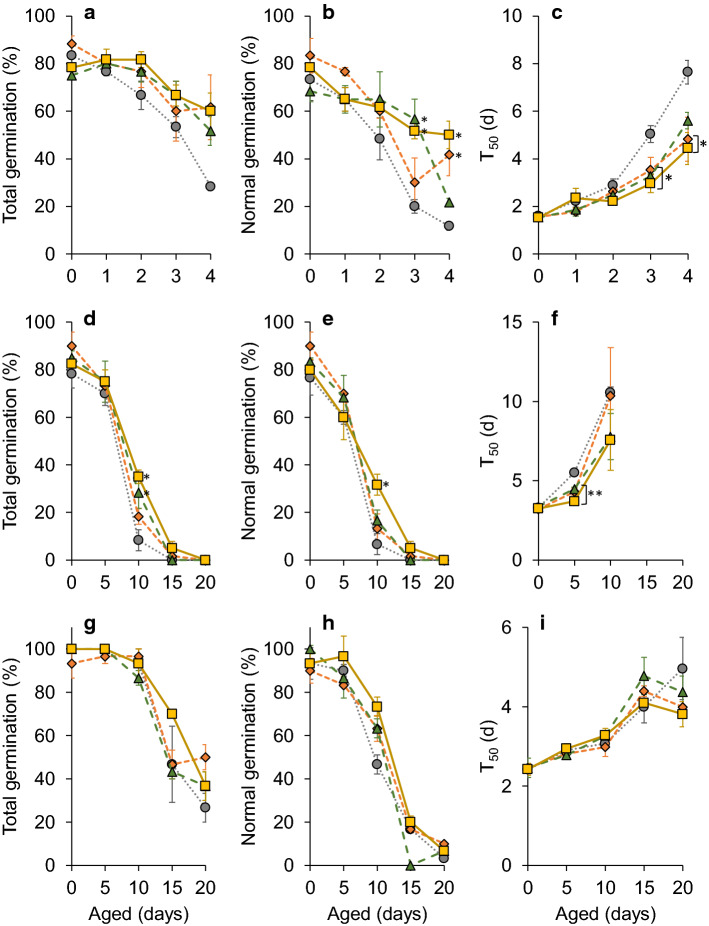
Total germination, normal germination and time taken for germination to reach 50% (T_50_) for *A. githago* (**a–c**), *L. perenne* (**d–f**) and *P. sativum* (**g–i**) seeds following ageing at 50 °C and 60% RH. The coloured symbols/lines represent different treatments: control (grey circles and dotted line), seeds aged in the presence of O_2_ absorbers (orange diamonds and short dashed line), silica gel (green triangles and long dashed line) or O_2_ absorbers and silica gel (yellow squares and solid line). Values are means of 3 replicates ± SE. Asterisks denote significant differences (*, *P* < 0.05; **, *P* < 0.01) between treated and control seeds at a particular ageing time point determined using one-way ANOVA

Total germination, assessed as radicle emergence, of all three species declined during artificial ageing. Control *A. githago* seeds had initial total germination of 83 ± 4.41%, but after 4 d of ageing total germination decreased to 28 ± 1.67%. Seeds aged in the presence of O_2_ absorbers did not show such a large decline in germination, and after 4 d of ageing, total germination was 62 ± 13.64%. Silica also reduced loss of germinability, with total germination of 52 ± 6.01% after 4 d of ageing. However, the differences between the treatments were not statistically significant (Fig. 3a). Similar patterns were observed for *L. perenne*, where total germination was significantly (*P* < 0.05) higher in seeds aged for 10 d in the presence of silica and O_2_ absorbers + silica compared to control seeds (Fig. 3d). Likewise, O_2_ absorbers and silica treatments reduced the decline in germination during artificial ageing of *P. sativum* seeds, albeit not significantly (Fig. 3g).

Normal germination followed a similar pattern to total germination and was significantly (*P* < 0.05) higher in *A. githago* seeds aged in the presence of silica and O_2_ absorbers + silica for 3 d, and O_2_ absorbers and O_2_ absorbers + silica for 4 d compared to control seeds (Fig. 3b). *L. perenne* also showed significantly (*P* < 0.05) higher normal germination of seeds aged with O_2_ absorbers + silica for 10 d compared to control seeds (Fig. 3e).

The rate of germination, measured as the time taken for 50% of seeds to germinate (T_50_), increased as ageing progressed e.g. T_50_ increased from 1.6 ± 0.15 d in unaged control *A. githago* seeds to 7.6 ± 0.50 d in seeds aged for 4 d. The increase in T_50_ was significantly (*P* < 0.05) lower for seeds aged for 3 or 4 d in the presence of O_2_ absorbers and/or silica (Fig. 3c). *L. perenne* seeds aged for 5 d with O_2_ absorbers and/or silica also had significantly (*P* < 0.01) lower T_50_ compared to control seeds (Fig. 3f). There were no significant differences in T_50_ between treatments for *P. sativum* seeds (Fig. 3i).

The longevity of the three species during artificial ageing was calculated using Probit analysis to determine the time taken for half of the seeds to lose viability (*P*_50_). The *P*_50_ of control seeds ranged from 2.8 d for *A. githago* to 16 d for *P. sativum*. Treatment with O_2_ absorbers, silica and O_2_ absorbers + silica significantly increased *P*_50_ for *A. githago* to 4.8, 5.2 and 5.5 d, respectively. Treatment with O_2_ absorbers + silica also increased *P*_50_ of *L. perenne* seeds from 5.1 to 10.7 d. In contrast, the *P*_50_ of *P. sativum* was unaffected by the treatments (Table 2).

**Table 2:** Time taken for total germination to fall to 50% of initial germination (*P*_50_) determined using Probit analysis

	*P*_50_ (d)			
	Control	O_2_ absorbers	Silica	O_2_ ab. + Silica
*A. githago*	2.843 (2.481–3.303)	**4.815 (3.444–12.59)**	**5.159 (3.799–10.38)**	**5.545 (4.094–10.89)**
*L. perenne*	5.106 (3.563–6.517)	6.563 (5.529–7.572)	6.714 (5.312–8.051)	**10.65 (8.835–12.66)**
*P. sativum*	16.08 (14.32–18.25)	18.75 (15.09–27.89)	16.35 (14.95–18.08)	18.01 (16.81–19.59)

Values in parentheses represent lower and upper 95% confidence intervals. Values in bold are significantly different compared to the control seeds based on non-overlapping confidence intervals

### Effect of storage environment on volatile profiles

**Fig. 4:**
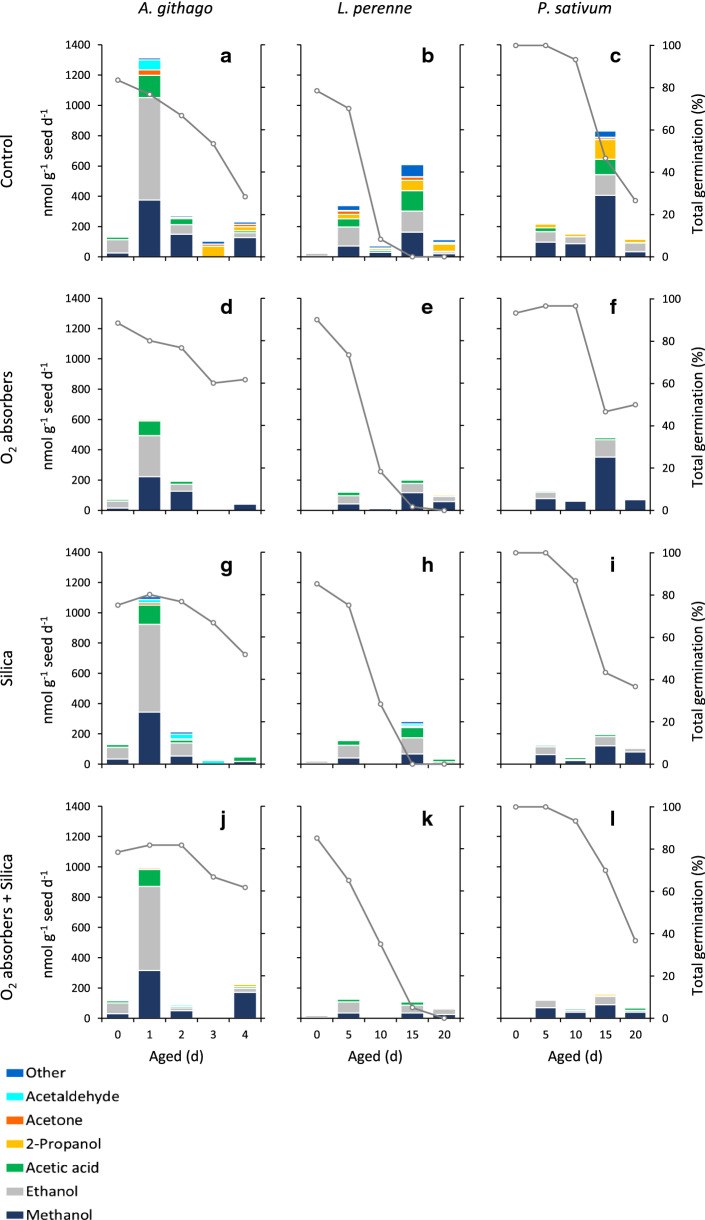
Stacked bars showing the rate of volatile emission during ageing of seeds of *Agrostemma githago*, *Lolium perenne*, and *Pisum sativum* under control conditions (**a**–**c**), in the presence of O_2_ absorbers (**d**–**f**), in the presence of silica gel (**g**–**i**), and in the presence of O_2_ absorbers and silica gel (**j**–**l**). The overall height of the bars corresponds to the mean total volatile emission (*n* = 3), whilst the coloured bands represent individual volatile compounds. Volatile emission was calculated as nmol g^−1^ seed d^−1^ on a rolling basis throughout the ageing time course, in that at each ageing time point, the amounts of each volatile accumulated at the previous time point were subtracted, and the remaining amount was divided by the number of days of ageing between sampling intervals. The line plots represent mean total germination (*n* = 3) under each of the conditions

Total volatile emission, calculated as the sum of the amount of identified and quantified volatiles, from unaged control seeds ranged from 368 nmol g^−1^ FW for *P. sativum* up to 658 nmol g^−1^ FW for *L. perenne*. In all three species, volatile emission was higher from aged seeds but did not follow a consistent increase as ageing progressed. Volatile emission from *A. githago* peaked at 1344 nmol g^−1^ FW after 2 d of ageing, representing around a 2.5-fold increase compared to unaged seeds. In contrast, volatile emission increased over eightfold between 0 and 20 d aged *L. perenne* seeds, and over 16-fold between 0 and 15 d aged *P. sativum* seeds (Table 3). However, when the rate of total volatile emission per day of ageing was calculated after subtraction of the total volatiles detected at the previous time point, the highest rate of volatile emission was similar for *A. githago* and *P. sativum* seeds at around 800 nmol g^−1^ seed day^−1^. For unaged controls, seeds were incubated at 20 °C for the duration of the ageing time course (4 d for *A. githago* and 20 d for *L. perenne* and *P. sativum*), and the total volatile emission rate was calculated by dividing the total volatile emission by the number of days of incubation at 20 °C. Ageing seeds in the presence of O_2_ absorbers generally decreased volatile emission by around twofold compared to control seeds. Silica and O_2_ absorbers + silica had little effect on volatile emission from *A. githago* seeds but did significantly reduce volatile emission from *L. perenne* and *P. sativum* seeds, by around two- to fourfold at the later ageing time points (Fig. 4).

**Table 3:** Total volatile emission of *Agrostemma githago*, *Lolium perenne* and *Pisum sativum* seeds during ageing at 50 °C and 60% RH determined using SPME–GC–MS

Aged (d)	Total volatile emission (nmol g^−1^ FW)							
	Control		O_2_ absorbers		Silica		O_2_ ab. + Silica	
*A. githago*								
0	545.4	ab	299.3	a	547.6	ab	475.4	ab
1	1313.4	f	597.9	abcd	1106.2	ef	993.6	cdef
2	1343.5	f	793.1	bcde	1303.7	f	985.7	cdef
3	1000.8	def	499.4	ab	776.9	bcde	560.5	abc
4	1190.6	ef	519.0	ab	621.8	abcd	783.2	bcde
*L. perenne*								
0	658.0	ab	310.8	a	511.5	a	416.7	a
5	1689.4	cde	623.7	ab	880.1	abc	642.9	ab
10	1962.0	de	681.2	ab	613.2	ab	611.5	ab
15	5005.0	f	1721.4	cde	2018.7	e	1153.7	abcd
20	5368.6	f	2242.4	e	1932.8	de	1456.6	bcde
*P. sativum*								
0	367.9	ab	243.6	a	280.5	ab	268.8	ab
5	1167.3	abc	646.6	abc	642.7	abc	621.1	ab
10	1918.2	abcde	979.1	abc	863.1	abc	933.1	abc
15	6062.4	g	3394.8	de	1928.3	abcde	1744.8	abcd
20	5275.8	fg	3605.7	ef	2396.0	cde	2039.7	bcde

Values are mean ± SE (*n* = 3). Letters indicate significant differences (*P* < 0.05) between treatments and ageing time points determined using two-way ANOVA and Tukey’s post hoc test

Twenty-four different volatile compounds were detected across the 3 species, including a range of alcohols, aldehydes, ketones and furans (Supplementary Fig. S1). However, the volatile profiles of all three species were dominated by ethanol, methanol, acetic acid and 2-propanol (Fig. 4). Ageing in the presence of O_2_ absorbers and/or silica tended to reduce the emission of individual volatiles. O_2_ absorbers were more effective than silica at reducing the emission of ethanol, acetic acid and acetone, whilst silica was more effective at reducing emission of methanol, particularly in *L. perenne* and *P. sativum* seeds (Fig. 4).

**Fig. 5:**
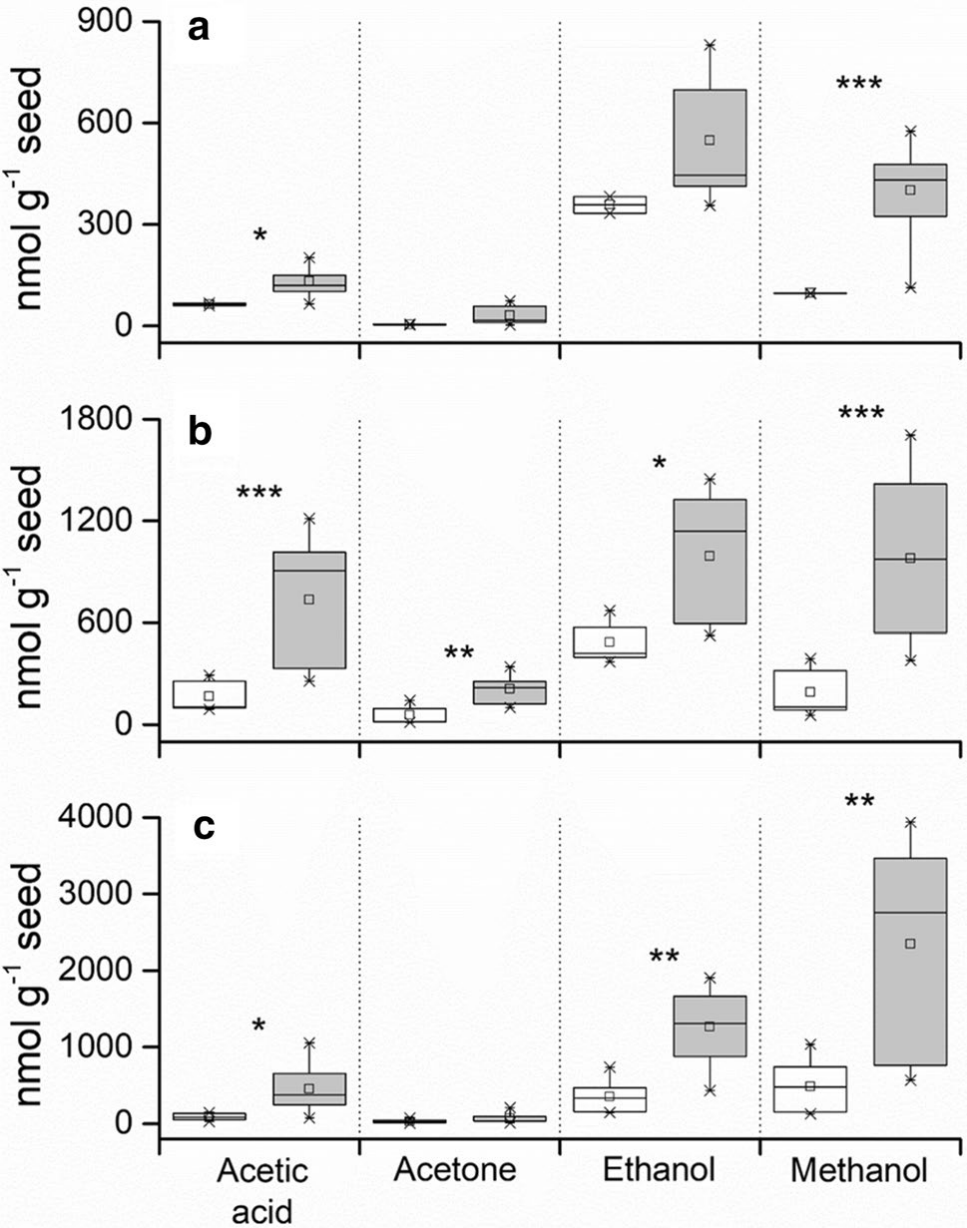
Box plots showing emission of acetic acid, acetone, ethanol and methanol during ageing of control seeds of *Agrostemma githago* (**a**), *Lolium perenne* (**b**), *Pisum sativum* (**c**). White boxes represent seeds with > 85% total germination (> 70% for *Lolium perenne*), and grey boxes represent seeds with < 85% total germination (< 70% for *Lolium perenne*). The boxes span the 25^th^ to 75^th^ percentiles, with the median and mean represented by a line and square, respectively. Whiskers show the minimum and maximum values. Asterisks indicate significant differences (*, *P* < 0.05; **, *P* < 0.01; ***, *P* < 0.001) in the emission of individual volatiles from seeds with high (> 85% or 70%) and low (< 85% or 70%) germination determined using a two-sample, two-tailed *t*-test

Volatile emission increased as seed germination decreased, and for acetic acid and methanol emission was significantly higher from samples with total germination less than 85% for *A. githago* (47/60 samples) and *P. sativum* (28/60 samples) and less than 70% for *L. perenne* (40/60 samples). There was also significantly higher emission of ethanol from seeds of *L. perenne* and *P. sativum* with total germination less than 70% and 85%, respectively. Whilst acetone showed significantly higher emission only from seeds of *L. perenne* with total germination less than 70% (Fig. 5).

PCA based on 26 VOC and germination variables separated the three species and the four storage treatments. The first four factors explained 70% of the observed variation (Suppl. Table S2). The first factor (F1) accounted for 36% of the variation. The variables with the highest loading in F1 were methyl acetate, 2-methyl-1-propanol, 2-methyl-1-butanol, 1-butanol, 2-butanol, 2-pentanone, 2-ethylfuran and 2-methylfuran. The second factor (F2) accounted for 17% of the variation, and total VOC, ethanol, methanol, and acetic acid emission were the variables with the highest loading in F2 (see Supplementary Fig. S2). F3 and F4 explained 10% and 7% of the observed variation, respectively. Total germination and normal germination showed the greatest correlation with F4 (Table 4). Species and treatments were projected as supplementary variables onto the PCA space defined by F1 and F2. F1 separated the control treatment from the other three treatments, with the control treatment centred towards greater VOC emission. *A. githago* showed the greatest separation from *L. perenne* in F2 (Supplementary Fig. S2). This corresponded to greater emission of acetaldehyde and ethanol from *A. githago* seeds aged for 1 d in the control and Silica treatments, whilst aged, control *L. perenne* seeds with declining or no germinability were characterised by higher emission of furans, branched alcohols, 2-butanone and 2-pentanone.

**Table 4:** Correlations between 26 variables and the first 6 factors (PCA axes) for germination and VOC emission for *Agrostemma githago*, *Lolium perenne* and *Pisum sativum* across all 4 storage treatments

Variables	F1	F2	F3	F4	F5	F6
Total germination	− 0.336	0.276	− 0.199	**0.813**	0.040	− 0.041
Normal germination	− 0.315	0.182	− 0.288	**0.798**	0.018	− 0.097
Acetaldehyde	0.112	0.447	− 0.251	− 0.135	− 0.121	− 0.342
Dimethylsulfide	0.060	− 0.152	0.027	− 0.537	− 0.276	− 0.168
Propanal	0.561	0.331	0.587	0.155	− 0.003	0.178
Acetone	0.520	0.327	− 0.264	− 0.045	0.503	− 0.163
Methyl acetate	**0.853**	− 0.194	0.114	− 0.082	0.221	0.123
2-Methylfuran	**0.744**	− 0.447	− 0.216	− 0.030	0.121	− 0.145
Ethyl acetate	0.212	− 0.081	− 0.087	− 0.217	0.775	− 0.192
Methanol	0.439	**0.746**	− 0.084	− 0.094	− 0.051	0.014
2-Butanone	0.692	− 0.362	− 0.283	0.063	0.290	− 0.119
2-Methylbutanal	0.532	0.343	0.571	0.099	− 0.035	− 0.339
3-Methylbutanal	0.584	0.352	0.600	0.118	− 0.054	− 0.276
2-Propanol	**0.708**	0.142	0.555	0.075	0.043	0.057
Ethanol	0.308	**0.763**	− 0.462	− 0.086	− 0.126	− 0.132
2-Ethylfuran	**0.770**	− 0.366	− 0.039	0.069	− 0.360	− 0.168
2-Pentanone	**0.783**	− 0.397	− 0.282	0.161	− 0.112	− 0.065
Acetonitrile	0.130	0.436	− 0.439	− 0.217	− 0.226	0.397
2-Butanol	**0.772**	0.114	0.369	0.177	− 0.019	0.299
1-Propanol	0.578	0.343	− 0.027	− 0.118	0.117	0.302
2-Methyl-1-propanol	**0.844**	− 0.318	− 0.118	0.100	− 0.055	0.120
1-Butanol	**0.804**	− 0.352	− 0.279	0.162	0.037	0.146
2-Methyl-1-butanol	**0.821**	− 0.376	− 0.204	0.111	− 0.111	0.167
2-Pentylfuran	0.570	− 0.414	− 0.123	0.018	− 0.445	− 0.306
Acetic acid	0.647	0.622	− 0.267	0.002	− 0.052	0.040
Total	0.528	**0.779**	− 0.262	− 0.078	− 0.072	-0.064
Eigenvalue	9.241	4.468	2.662	1.913	1.548	1.047
Variability (%)	35.542	17.183	10.238	7.358	5.956	4.029
Cumulative %	35.542	52.725	62.963	70.321	76.277	80.306

Values in bold indicate variables with a high contribution to each factor. The eigenvalues, variability and cumulative variability for each factor are shown in the lower part of the table

### Influence of volatile accumulation in the headspace on seed storage longevity

**Fig. 6:**
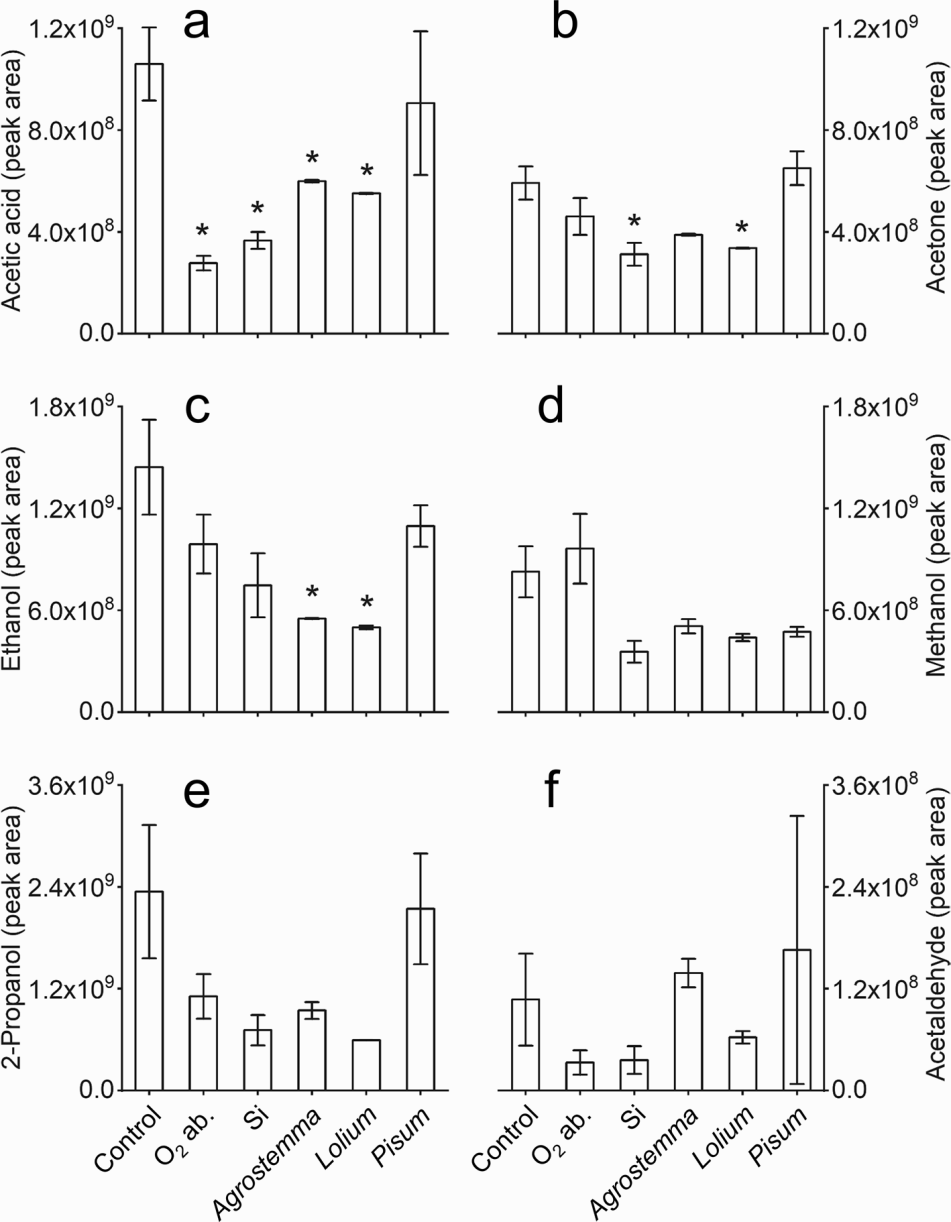
The effect of oxygen absorbers, silica gel and *P. sativum* seeds on the detection of acetic acid (**a**), acetone (**b**), ethanol (**c**), methanol (**d**), 2-propanol (**e**) and acetaldehyde (**f**) in the headspace of 20 mL vials containing 0.6 μmol acetic acid, 90 nmol acetone, 1.25 μmol ethanol, 3 μmol methanol, 0.85 μmol 2-propanol, or 40 nmol acetaldehyde. Vials either contained only the volatile compound (control), or the volatile compound in addition to an O_2_ absorbing sachet (O_2_ ab.), a silica gel sachet (Si; equilibrated to 60% RH) or 1 g of *A. githago*, *L. perenne* or *P. sativum* seeds that had been equilibrated to 60% RH. All vials were incubated at 50 °C for 10 d. Bars represent the mean volatile emission ± SE. (*n* = 3). Asterisks denote significant differences (*P* < 0.05) in volatile abundance between the control vials and those containing O_2_ absorbers, silica or *P. sativum* seeds determined using one-way ANOVA and a post hoc Dunnett’s test

The adsorption of volatiles by O_2_ absorbers, silica, *A. githago*, *L. perenne* and *P. sativum* seeds was tested by comparing the abundance of acetic acid, acetone, ethanol, methanol, 2-propanol and acetaldehyde after 10 d incubation at 50 °C. Significantly lower levels of acetic acid were detected in vials containing O_2_ absorbers, silica, *A. githago* and *L. perenne* seeds (*P* < 0.05; Fig. 6a) whilst silica and *L. perenne* seeds significantly reduced the abundance of acetone (*P* < 0.05; Fig. 6b), and levels of ethanol were significantly reduced by *A. githago* and *L. perenne* seeds (*P* < 0.05; Fig. 6c). There were no significant effects on the abundance of the other volatile compounds, although methanol abundance appeared to be reduced by silica and seeds of all three species; and O_2_ absorbers, silica, *A. githago* and *L. perenne* seeds appeared to reduce levels of 2-propanol (Fig. 6).

To test the effects of individual volatile compounds on seed germinability, *P. sativum* seeds were aged for 10 d in the presence of acetaldehyde, acetic acid, acetone, ethanol, methanol or 2-propanol. Only acetone and methanol had a significant effect, reducing total germination from 77% in 10 d aged controls to 43% and 20%, respectively (*P* < 0.05; Table 5).

**Table 5:** Total germination of *Pisum sativum* seeds after ageing for 0 or 10 days at 50 °C and 60% RH in the presence of either a 20 cc oxygen absorber (O_2_ absorber), silica gel sachet, acetaldehyde, acetic acid, acetone, ethanol, methanol or 2-propanol

	Aged (d)	Total germination (%)	
Control	0	100.0 ± 3.33	
O_2_ absorbers	0	100.0 ± 4.08	
Silica	0	93.3 ± 2.33	
Control	10	76.7 ± 0.00	c
O_2_ absorbers	10	76.7 ± 14.53	c
Silica	10	73.3 ± 12.02	bc
Acetaldehyde	10	80.0 ± 18.56	c
Acetic acid	10	66.7 ± 13.33	bc
Acetone	10	43.3 ± 1.11	ab
Ethanol	10	60.0 ± 8.16	bc
Methanol	10	20.0 ± 5.88	a
2-Propanol	10	66.7 ± 13.47	bc

Values are means ± SE of 3 replicates. Different letters indicate significant differences (*P* < 0.05) between treatments for seeds aged for 10 d determined using ANOVA and a post hoc Tukey’s test

## Discussion

The three species are phylogenetically distant, *L. perenne* belongs to the Poaceae family within the monocotyledons, *P. sativum* belongs to Leguminosae within the Rosid clade and *A. githago* belongs to the Caryophyllaceae family within the order Caryophyllales, which is considered to be sister to the Asterids. They also differ in seed structure; the seeds of both *L. perenne* and *A. githago* are endospermic, whilst those of *P. sativum* are non-endospermic (Fig. 1). Endospermic seeds have been reported to be shorter lived than non-endospermic seeds (Probert et al. 2009) and the results obtained in this study follow this pattern with seeds of *P. sativum* being the longest lived with a *P*_50_ of 16 d compared to 2.8 d and 5 d for *A. githago* and *L. perenne*, respectively (Table 2). Similarly, *P. sativum* was classified as long-lived under ambient (*P*_50_ = 15.9 y) and cold (*P*_50_ = 97 y) storage conditions, whilst *L. perenne* was classified as medium-lived with *P*_50_ values of 7.2 y and 41 y under ambient and cold storage conditions, respectively (Colville and Pritchard 2019).

Despite the differences in phylogeny and seed structure, the volatile profiles of the three species were very similar, with the major volatiles being methanol, ethanol, acetic acid, 2-propanol and acetone. Methanol, ethanol and acetone have also been widely reported in other studies of seed volatile production during storage (Buckley and Buckley 2009; Mira et al. 2010, 2016; Colville et al. 2012). This indicates that the major volatile compounds are derived from core processes occurring in seeds during artificial ageing, and probably not greatly influenced by the chemical composition or structure of seeds. However, the oil content of the seeds of all three species was fairly low at 0.7% for *P. sativum*, 1.5% for *L. perenne*, and 4.4% for *A. githago*, as determined by time domain-nuclear magnetic resonance (TD-NMR), so the seeds are dominated by starchy reserves. Although lower in abundance, VOCs likely to derive from lipid peroxidation, such as alcohols (2-methyl-1-propanol, 2-methyl-1-butanol, and 2-propanol, etc.), ketones (2-butanone and 2-pentanone), and 2-methylfuran and 2-pentylfuran showed a significant, albeit weak negative correlation with germination (Supplementary Table S1). For oily seeds, it might be expected that a greater proportion of volatiles deriving from lipid peroxidation e.g. aldehydes would be emitted, although fermentation-type reactions have been shown to dominate in seeds with 10—32% oil content stored under humid conditions (75% RH) compared to drier (< 33% RH) conditions (Mira et al. 2016). Based on the mean seed moisture content and oil content of the seeds used in this study, the eRH at the elevated temperature of ageing (50 °C) was estimated using Royal Botanic Gardens, Kew’s Seed Information Database (2020). The eRH was estimated to be 76, 61 and 69% for *A. githago*, *L. perenne*, *P. sativum*, respectively, which means that during ageing at 50 °C the seeds are close to or just over the eRH threshold at which mitochondrial respiration can occur.

Storage of seeds under anoxia has been shown to increase longevity. Celery and celeriac seeds stored in sealed jars at 35 °C for 17 d with an oxygen absorber and zeolite Drying Beads™ showed 97% and 85% normal germination, respectively, compared to 0% and 23% when stored without oxygen absorbers or Drying Beads™ (Groot et al. 2015). In that experiment, storage with either an oxygen absorber or Drying Beads™ alone did not improve longevity, which was likely due to the moisturisation effect of the oxygen absorbers, and the ultra-drying effect of the Drying Beads™, respectively. In this study, neither the oxygen absorbers nor silica gel had any significant effect on the moisture content of the seeds (Table 1). This suggests that the moisturising agent in the O-Buster O_2_ absorbers did not release sufficient excess water to affect the moisture content of the seeds, which had been equilibrated at 60% RH prior to ageing. Perhaps this could be an issue for seeds at lower moisture content, and in this case, alternative oxygen absorbers, which function at low RH without requiring water for absorption of oxygen should be used. The silica gel had no drying effect on the seeds because it had been equilibrated at 60% RH along with the seeds prior to the ageing experiments. The incubation of silica gel with O_2_ absorbers had no effect on the eRH of the silica gel, confirming that the O_2_ absorbers did not cause measurable changes in the moisture status within the sealed vials. The life span (*P*_50_) of *A. githago* seeds was significantly increased by the presence of O_2_ absorbers (69% increase), silica (81% increase) and O_2_ absorbers + silica (95% increase), whilst that of *L. perenne* was increased by O_2_ absorbers + silica (108% increase; Table 2). This indicates that the positive effects of the treatments on life span are not solely due to reduction of oxygen levels. Volatile emission during ageing was monitored, and it was shown that ageing in the presence of O_2_ absorbers and/or silica tended to reduce total volatile emission by around twofold, particularly at the later ageing time points (Table 3). Since none of the treatments had any effect on seed moisture content, and silica treatment did not influence oxygen levels in the storage containers it is likely that the reduction in the abundance of volatiles detected is at least partly due to adsorption of seed-derived volatiles by the silica gel and possibly also the O_2_ absorbers. This was tested by incubating the silica gel sachets and O_2_ absorbers in sealed vials containing either methanol, ethanol, acetic acid, acetone, 2-propanol, or acetaldehyde at 50 °C for 10 d. There was a fairly high degree of variability in the data, probably because of the volatility of compounds and therefore the difficulty of minimising losses during preparation of the samples. However, significant reduction of acetic acid by O_2_ absorbers and silica, and acetone by silica, was observed. Although not significant, 2-propanol levels appeared to be reduced by O_2_ absorbers, and 2-propanol and methanol were detected at lower levels in the presence of silica (Fig. 6). This indicates that silica, and O_2_ absorbers to a lesser degree, could adsorb volatiles released by seeds during artificial ageing. The O_2_ absorbers contain zeolites, which along with silica gel have been found to adsorb VOCs. A comparison of the adsorption of a range of VOCs by silica gel, zeolites and activated charcoal showed that silica gel was the most adsorbent overall, and acetic acid was the VOC that was most strongly adsorbed by silica gel and zeolites (McGath et al. 2014).

Adsorption of exogenously applied volatile compounds by seeds was tested by incubating *A. githago*, *L. perenne* and *P. sativum* seeds with methanol, ethanol, acetic acid, acetone, 2-propanol or acetaldehyde at 50 °C for 10 d. *A. githago* and *L. perenne* seeds significantly reduced the levels of acetic acid and ethanol, and *L. perenne* also reduced the levels of acetone. There were no significant reductions in the abundance of volatiles detected in the presence of *P. sativum seeds* (Fig. 6). This implies that for *P. sativum* seeds under the conditions used in this study, adsorption of volatiles by the seeds is not a major factor contributing to viability loss. Although, it is possible that seeds are re-adsorbing some of the volatiles released during ageing and reaching saturation, preventing further adsorption of exogenously applied volatile compounds. The adsorption of acetic acid, ethanol and acetone by *A. githago* and/or *L. perenne* seeds, but not *P. sativum* seeds, could explain why the O_2_ absorber and silica gel treatments only had a beneficial effect on the lifespan of *A. githago* and *L. perenne* seeds. Mira et al. (2016) showed that the levels of volatiles released by mixtures of seeds were not equal to what would be expected from the sum of volatile profiles of the individual species, and proposed that this indicated that the seeds were adsorbing volatiles from the headspace of the storage container. However, they found no detrimental effect on longevity as a result of volatile adsorption, so concluded that volatile accumulation during seed storage did not contribute to seed deterioration.

In this study, the total germination of *P. sativum* seeds artificially aged for 10 d in the presence of exogenously applied acetone or methanol was significantly decreased compared to control seeds, but ethanol, acetic acid, 2-propanol and acetaldehyde had no effect. This suggests that some seed-derived compounds including acetone and methanol may be toxic and accumulation within seed storage containers has the potential to shorten seed longevity. Acetone was also shown to be toxic when pea seeds were soaked in acetone and then subjected to accelerated ageing (100% RH and 40 °C for 4 d) or controlled deterioration (20% MC, 40 °C for 4 d). Susceptibility was dependent on MC and was more apparent after ageing, particularly in seeds subjected to controlled deterioration (Coolbear et al. 1991). However, the toxicity of volatile compounds may be species-dependent and influenced by storage conditions. Zhang et al. (1994) also reported that some exogenously applied volatile compounds were toxic to seeds, but in contrast to our results, they found that methanol had no deleterious effects, whilst acetone reduced germination of rice seeds and soybean seeds stored at 23 °C, but had no effect on lettuce or sunflower seeds. In their study, acetaldehyde and acetic acid were the most toxic across all storage conditions and all four species, ethanol was toxic only at higher RH conditions, and 2-propanol only affected rice and soybean seeds. They applied the volatile treatments at a concentration of 4 mM for all except acetaldehyde which was 1 mM. In contrast, the concentrations used in this study ranged from 2 μM for acetaldehyde to 150 μM for methanol. We attempted to use physiologically relevant concentrations of volatile compounds, i.e., treatments were applied at levels similar to those released by *P. sativum* seeds with moisture content of 11.9% during ageing at 50 °C, so the ‘naturally’ low concentrations used could explain why we did not observe any toxic effects of acetaldehyde, acetic acid, ethanol and 2-propanol.

The rate of volatile release from seeds depends upon the rate of volatile formation and the rate of diffusion from the site of formation to the exterior of the seed. The latter is determined by the properties of the seed matrix and the volatile compound and the diffusion distance. Oxygen diffusion can occur in cellular glasses, such as those found in dry seeds (El-Maarouf-Bouteau and Bailly 2008). Intercellular spaces are the main path for oxygen diffusion within seeds (Cloetens et al. 2006). Micro-CT images of a *P. sativum* seed (Fig. 1d and e) showed intercellular spaces, particularly in the cotyledons, indicating that gas diffusion via intercellular spaces may vary between different seed tissues.

Cuticular layers are a barrier to oxygen uptake/release (Borisjuk and Rolletschek 2009). Cuticles are a hydrophobic layer comprising cutin and cuticular waxes. Seeds may have multiple cuticles, which can be associated with the seed coat, and also surround the embryo and endosperm. They play a role in controlling water uptake/loss as well as gas diffusion (Ingram and Nawrath 2017). In developing seeds, the internal oxygen concentration is low (Borisjuk and Rolletschek 2009), and cuticles have been proposed to limit oxygen diffusion and thereby reactive oxygen species production to protect against oxidation and viability loss (De Giorgi et al. 2015). The cuticle also provides the greatest resistance to volatile emission (Widhalm et al. 2015). The composition and permeability of cuticles varies between species and organs and this may explain the different responses of the three species to ageing under hypoxia. The life span of *A. githago* seeds showed the greatest increase when aged with O_2_ absorbers, silica or O_2_ absorbers + silica. Perhaps the cuticle of *A. githago* is more permeable to oxygen and volatile compounds than that of *L. perenne* or *P. sativum*. This could explain the higher volatile emission rate from unaged *A. githago* seeds compared to unaged *L. perenne* and *P. sativum* seeds (Fig. 4a–c). Volatile emission from *A. githago* followed a different pattern to that of *L. perenne* and *P. sativum* seeds, and volatile emission rate was highest after 1 d of ageing, it then fell to its lowest level after 3 d of ageing before rising slightly after 4 d ageing. In contrast, *L. perenne* and *P. sativum* both showed a biphasic pattern of volatile emission, with an initial small increase in volatile emission rate after 5 d of ageing and then a larger increase after 15 d of ageing (Fig. 4). The peak in volatile emission rate after 15 d of ageing coincided with the onset of rapid viability loss in *P. sativum* and almost complete loss of viability in *L. perenne* (Fig. 3d and g)*.* This could indicate that barriers to gas diffusion, e.g., cuticles were degraded during ageing leading to the large increase in volatile emission. The seeds were aged in sealed headspace vials and volatile analysis measured the accumulation of volatiles over the ageing time course. Therefore, decreases in volatile abundance indicate that the volatile compounds were being re-adsorbed by the seeds. In *P. sativum* seeds gas exchange is restricted mainly to the micropylar region (Wager 1974), although in dry *P. sativum* seeds the hilar slit is open (Fig. 1e) and may also be a path for gas diffusion. The seeds are also larger (Fig. 1) so diffusion of oxygen and volatile compounds into and out of the seeds will likely take longer than for *A. githago* and *L. perenne* seeds.

These experiments reveal that seed ageing can be slowed for some species by storage of seeds in an oxygen-free atmosphere. Furthermore, the volatile compounds released by seeds during storage may be re-adsorbed by seeds leading to toxic effects and loss of viability. Therefore, volatile traps such as silica gel may also be beneficial for extending seed life span in storage. There is variation between species in terms of their response to oxygen and in their temporal patterns of volatile emission, which may relate to differences in gas permeability due to seed size, structure and the presence of diffusion barriers such as cuticles. Future work will assess the influence of these seed traits on the potential for storage under anoxia to extend seed life span. Cuticles are considered to be the main barrier to oxygen diffusion, but the permeability of cuticles increases markedly at temperatures above 35 °C (Schreiber et al. 2001; Riederer 2006), so under the artificial ageing conditions used in this study the effect of any inter-specific differences in cuticle composition and permeability may be less marked than under lower temperature storage conditions. Therefore, the extent to which anoxia may extend seed life span may be even greater at lower temperatures such as those used for commercial storage (c. 12 °C) or gene bank storage (-20 °C).

### Author contribution statement

Conceptualization: LC; Data curation: LC; Formal analysis: LC; Investigation: BH, VF, LC; Visualisation: LC; Writing–original draft: LC; Writing–review and editing: BH, VF, HWP, LC.

## Supplementary Information

Below is the link to the electronic supplementary material.

Supplementary file1 (PDF 461 KB)

## Data Availability

The data that support the findings of this study are available from the corresponding author upon request.

## Abbreviations

eRH: Equilibrium relative humidity
VOC: Volatile organic compound
