# Supplementary material for: Gaseous environment modulates volatile emission and viability loss during seed artificial ageing
Source: Planta. 2021 Apr 17;253(5):106. doi: 10.1007/s00425-021-03620-5 (PMC8053187; doi:10.1007/s00425-021-03620-5)
Supplement: Supplementary file 1 — Supplementary file1 (PDF 461 KB) [file 425_2021_3620_MOESM1_ESM.pdf]

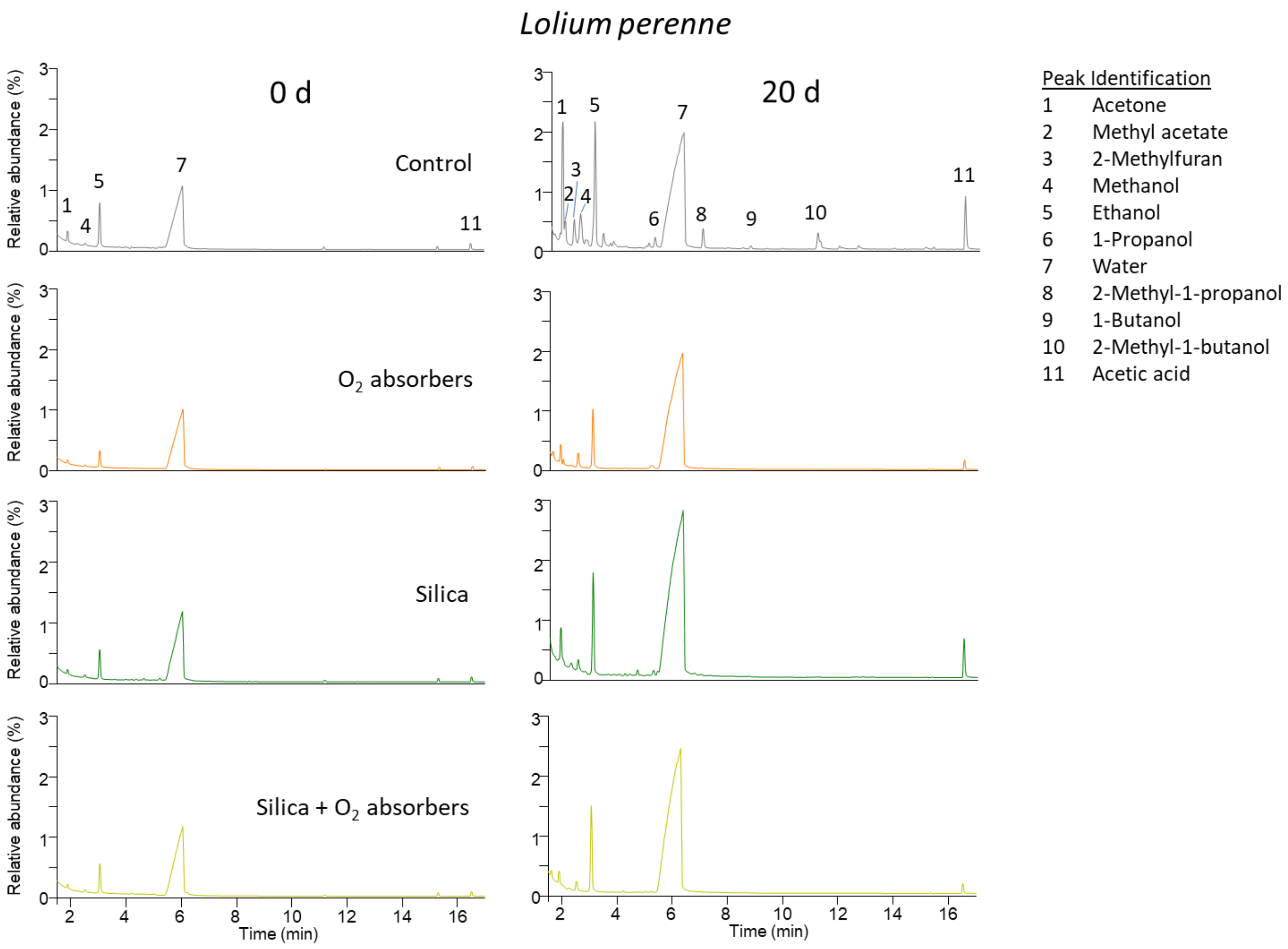

Suppl. Fig. S1 Example GC chromatograms of the headspace within vials containing *Lolium perenne* seeds at 60% RH after 20 d storage at 20°C (0 d) or 50°C (20 d). The headspace was sampled using solid phase microextraction (SPME). The treatments: control, O_2 absorbers, silica and silica + O_2 absorbers were as described in the main article. Numbered peaks correspond to the compounds in the peak identification list, which were identified by spectral library matching with the NIST database, and confirmed with analytical standards.

a

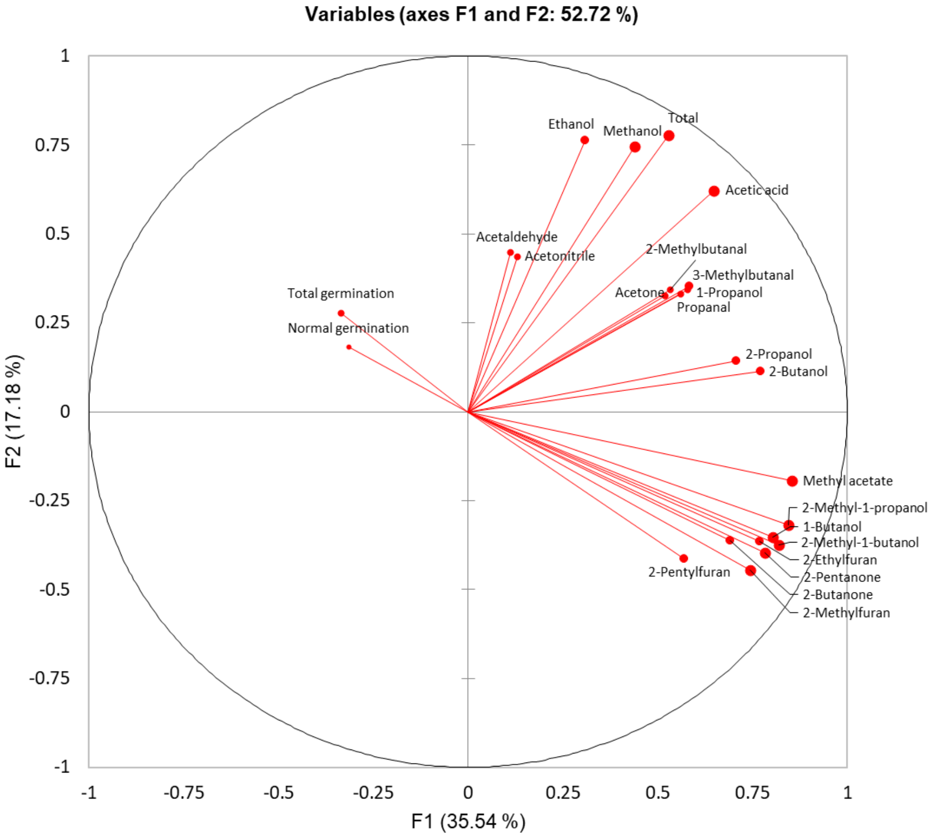

b

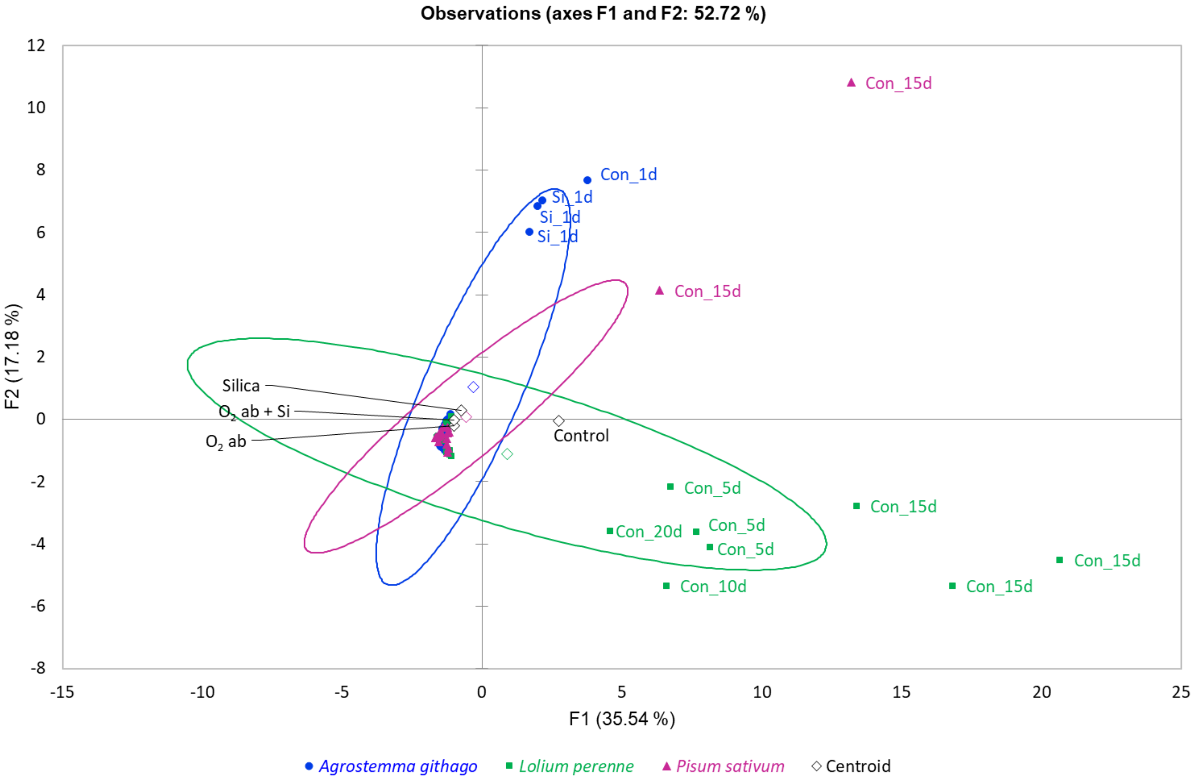

Suppl. Fig. S2 Plots from PCA of VOC emission and germination for all three species. Correlation circle showing the projection of 24 variables on axes F1 and F2 (**a**) and plot of observations in the PCA space coloured by species: *Agrostemma githago*, blue; *Lolium perenne*, green; *Pisum sativum*, magenta. The ellipses represent the 95% confidence intervals for each species (**b**).

Suppl. Table S1 Pearson correlation matrix of VOC emission and germination for *Agrostemma githago*, *Lolium perenne*, and *Pisum sativum*. Values in bold are different from 0 with a significance level alpha=0.05

Correlation matrix (Pearson (n)):

Variables	Total germination	Normal germination	Acetaldehyde	Dimethyl sulfide	Propanal	Acetone	Methyl acetate	2-Methylfuran	Ethyl acetate	Methanol	2-Butanone	2-Methylbutanal	3-Methylbutanal	2-Propanol	Ethanol	2-Ethylfuran	2-Pentanone	Acetonitrile	2-Butanol	1-Propanol	2-Methyl-1-propanol	1-Butanol	2-Methyl-1-butanol	2-Pentylfuran	Acetic acid	Total
Total germination	**1**	**0.893**	0.024	**-0.355**	-0.065	-0.037	**-0.412**	**-0.338**	**-0.169**	0.007	**-0.202**	-0.127	-0.126	**-0.200**	0.110	**-0.287**	**-0.193**	0.011	**-0.176**	**-0.154**	**-0.285**	**-0.199**	**-0.274**	**-0.258**	-0.041	0.019
Normal germination	**0.893**	**1**	0.007	**-0.268**	**-0.162**	-0.035	**-0.373**	**-0.256**	**-0.150**	-0.087	-0.132	**-0.162**	**-0.171**	**-0.273**	0.108	**-0.230**	-0.129	0.007	**-0.199**	**-0.193**	**-0.228**	**-0.147**	**-0.208**	**-0.184**	-0.026	-0.022
Acetaldehyde	0.024	0.007	**1**	-0.020	0.010	**0.179**	-0.031	-0.031	-0.028	**0.282**	-0.028	0.098	0.144	-0.016	**0.482**	-0.025	-0.028	**0.177**	0.001	**0.252**	-0.033	-0.025	-0.023	-0.023	**0.351**	**0.446**
Dimethylsulfide	**-0.355**	**-0.268**	-0.020	**1**	-0.025	-0.046	0.069	0.095	-0.018	-0.031	0.055	-0.033	-0.025	0.027	-0.013	0.144	0.056	0.053	-0.051	-0.021	0.034	-0.002	0.036	**0.170**	-0.074	-0.026
Propanal	-0.065	**-0.162**	0.010	-0.025	**1**	**0.252**	**0.440**	0.134	0.008	**0.452**	0.107	**0.607**	**0.702**	**0.780**	0.093	**0.303**	**0.166**	-0.009	**0.820**	**0.412**	**0.302**	**0.195**	**0.229**	0.096	**0.424**	**0.372**
Acetone	-0.037	-0.035	**0.179**	-0.046	**0.252**	**1**	**0.408**	**0.331**	**0.453**	**0.461**	**0.414**	**0.212**	**0.221**	**0.317**	**0.475**	**0.186**	**0.302**	0.123	**0.283**	**0.429**	**0.296**	**0.337**	**0.263**	0.087	**0.566**	**0.568**
Methyl acetate	**-0.412**	**-0.373**	-0.031	0.069	**0.440**	**0.408**	**1**	**0.720**	**0.339**	**0.202**	**0.642**	**0.393**	**0.453**	**0.668**	0.038	**0.600**	**0.634**	0.002	**0.685**	**0.469**	**0.788**	**0.711**	**0.751**	**0.385**	**0.389**	**0.254**
2-Methylfuran	**-0.338**	**-0.256**	-0.031	0.095	0.134	**0.331**	**0.720**	**1**	**0.294**	0.000	**0.862**	**0.169**	**0.180**	**0.367**	0.009	**0.698**	**0.765**	-0.027	**0.376**	**0.245**	**0.727**	**0.760**	**0.740**	**0.610**	**0.244**	0.107
Ethyl acetate	**-0.169**	**-0.150**	-0.028	-0.018	0.008	**0.453**	**0.339**	**0.294**	**1**	0.050	**0.329**	0.043	0.029	0.074	-0.023	0.024	0.094	-0.050	0.063	0.122	0.142	0.142	0.103	-0.013	0.043	0.042
Methanol	0.007	-0.087	**0.282**	-0.031	**0.452**	**0.461**	**0.202**	0.000	0.050	**1**	0.018	**0.379**	**0.403**	**0.334**	**0.742**	0.105	0.074	**0.362**	**0.394**	**0.423**	**0.155**	0.090	0.090	0.003	**0.744**	**0.909**
2-Butanone	**-0.202**	-0.132	-0.028	0.055	0.107	**0.414**	**0.642**	**0.862**	**0.329**	0.018	**1**	**0.158**	**0.157**	**0.320**	0.047	**0.525**	**0.783**	-0.035	**0.314**	**0.311**	**0.656**	**0.790**	**0.641**	**0.405**	**0.280**	0.135
2-Methylbutanal	-0.127	**-0.162**	0.098	-0.033	**0.607**	**0.212**	**0.393**	**0.169**	0.043	**0.379**	**0.158**	**1**	**0.966**	**0.686**	**0.225**	**0.295**	**0.186**	-0.083	**0.515**	**0.317**	**0.255**	**0.147**	**0.151**	**0.164**	**0.403**	**0.401**
3-Methylbutanal	-0.126	**-0.171**	0.144	-0.025	**0.702**	**0.221**	**0.453**	**0.180**	0.029	**0.403**	**0.157**	**0.966**	**1**	**0.764**	**0.217**	**0.331**	**0.208**	-0.073	**0.613**	**0.337**	**0.301**	**0.187**	**0.210**	**0.174**	**0.436**	**0.421**
2-Propanol	**-0.200**	**-0.273**	-0.016	0.027	**0.780**	**0.317**	**0.668**	**0.367**	0.074	**0.334**	**0.320**	**0.686**	**0.764**	**1**	0.042	**0.472**	**0.326**	-0.033	**0.751**	**0.509**	**0.452**	**0.362**	**0.390**	**0.271**	**0.346**	**0.320**
Ethanol	0.110	0.108	**0.482**	-0.013	0.093	**0.475**	0.038	0.009	-0.023	**0.742**	0.047	**0.225**	**0.217**	0.042	**1**	0.019	0.061	**0.552**	0.103	**0.341**	0.058	0.084	0.060	-0.017	**0.822**	**0.926**
2-Ethylfuran	**-0.287**	**-0.230**	-0.025	0.144	**0.303**	**0.186**	**0.600**	**0.698**	0.024	0.105	**0.525**	**0.295**	**0.331**	**0.472**	0.019	**1**	**0.795**	-0.005	**0.506**	**0.251**	**0.760**	**0.666**	**0.783**	**0.926**	**0.270**	**0.162**
2-Pentanone	**-0.193**	-0.129	-0.028	0.056	**0.166**	**0.302**	**0.634**	**0.765**	0.094	0.074	**0.783**	**0.186**	**0.208**	**0.326**	0.061	**0.795**	**1**	0.014	**0.438**	**0.306**	**0.795**	**0.902**	**0.836**	**0.706**	**0.339**	**0.172**
Acetonitrile	0.011	0.007	**0.177**	0.053	-0.009	0.123	0.002	-0.027	-0.050	**0.362**	-0.035	-0.083	-0.073	-0.033	**0.552**	-0.005	0.014	**1**	0.014	**0.351**	0.011	0.034	0.046	-0.011	**0.443**	**0.479**
2-Butanol	**-0.176**	**-0.199**	0.001	-0.051	**0.820**	**0.283**	**0.685**	**0.376**	0.063	**0.394**	**0.314**	**0.515**	**0.613**	**0.751**	0.103	**0.506**	**0.438**	0.014	**1**	**0.461**	**0.639**	**0.552**	**0.635**	**0.270**	**0.493**	**0.373**
1-Propanol	**-0.154**	**-0.193**	**0.252**	-0.021	**0.412**	**0.429**	**0.469**	**0.245**	0.122	**0.423**	**0.311**	**0.317**	**0.337**	**0.509**	**0.341**	**0.251**	**0.306**	**0.351**	**0.461**	**1**	**0.334**	**0.358**	**0.314**	0.103	**0.535**	**0.500**
2-Methyl-1-propanol	**-0.285**	**-0.228**	-0.033	0.034	**0.302**	**0.296**	**0.788**	**0.727**	0.142	**0.155**	**0.656**	**0.255**	**0.301**	**0.452**	0.058	**0.760**	**0.795**	0.011	**0.639**	**0.334**	**1**	**0.855**	**0.923**	**0.567**	**0.412**	**0.228**
1-Butanol	**-0.199**	**-0.147**	-0.025	-0.002	**0.195**	**0.337**	**0.711**	**0.760**	0.142	0.090	**0.790**	**0.147**	**0.187**	**0.362**	0.084	**0.666**	**0.902**	0.034	**0.552**	**0.358**	**0.855**	**1**	**0.930**	**0.479**	**0.384**	**0.202**
2-Methyl-1-butanol	**-0.274**	**-0.208**	-0.023	0.036	**0.229**	**0.263**	**0.751**	**0.740**	0.103	0.090	**0.641**	**0.151**	**0.210**	**0.390**	0.060	**0.783**	**0.836**	0.046	**0.635**	**0.314**	**0.923**	**0.930**	**1**	**0.605**	**0.369**	**0.190**
2-Pentylfuran	**-0.258**	**-0.184**	-0.023	**0.170**	0.096	0.087	**0.385**	**0.610**	-0.013	0.003	**0.405**	**0.164**	**0.174**	**0.271**	-0.017	**0.926**	**0.706**	-0.011	**0.270**	0.103	**0.567**	**0.479**	**0.605**	**1**	0.130	0.057
Acetic acid	-0.041	-0.026	**0.351**	-0.074	**0.424**	**0.566**	**0.389**	**0.244**	0.043	**0.744**	**0.280**	**0.403**	**0.436**	**0.346**	**0.822**	**0.270**	**0.339**	**0.443**	**0.493**	**0.535**	**0.412**	**0.384**	**0.369**	0.130	**1**	**0.904**
Total	0.019	-0.022	**0.446**	-0.026	**0.372**	**0.568**	**0.254**	0.107	0.042	**0.909**	0.135	**0.401**	**0.421**	**0.320**	**0.926**	**0.162**	**0.172**	**0.479**	**0.373**	**0.500**	**0.228**	**0.202**	**0.190**	0.057	**0.904**	**1**

Values in bold are different from 0 with a significance level alpha=0.05

Suppl. Table S2 Eigenvalues and corresponding factors of Principal Component Analysis of VOC emission and germination for *Agrostemma githago, Lolium perenne,* and *Pisum sativum*.

	F1	F2	F3	F4	F5	F6	F7	F8	F9	F10	F11	F12	F13	F14	F15	F16	F17	F18	F19	F20	F21	F22	F23	F24	F25
Eigenvalue	9.241	4.468	2.662	1.913	1.548	1.047	0.855	0.741	0.673	0.623	0.501	0.357	0.307	0.262	0.201	0.124	0.118	0.102	0.080	0.056	0.048	0.036	0.018	0.013	0.006
Variability (%)	35.542	17.183	10.238	7.358	5.956	4.029	3.289	2.850	2.587	2.395	1.928	1.374	1.181	1.008	0.774	0.477	0.453	0.392	0.308	0.214	0.185	0.138	0.069	0.049	0.023
Cumulative %	35.542	52.725	62.963	70.321	76.277	80.306	83.595	86.445	89.032	91.427	93.355	94.729	95.910	96.918	97.692	98.169	98.622	99.014	99.322	99.535	99.721	99.859	99.928	99.977	100.000
